# Parental Stress in Autistic Children with Poor Oral Hygiene: A Pilot Study to Develop and Validate a Measurement Scale

**DOI:** 10.3390/healthcare12222215

**Published:** 2024-11-06

**Authors:** Pablo López Alegría, Síbila Floriano Landim, Vidal Antonio Pérez Valdés, Natalia Martínez Escudero, Juliana Nunes Botelho, Braulio Henrique Magnani Branco, Francisca Villagrán, Cristian Sandoval, Déborah Cristina de Souza Marques, Tesifon Parrón Carreño, Manuel Martín González

**Affiliations:** 1 Escuela de Terapia Ocupacional, Facultad de Psicología, Universidad de Talca, Talca 3465548, Chile; pablo.lopez@utalca.cl (P.L.A.); sibila.floriano@utalca.cl (S.F.L.); 2 Graduate Program in Health Promotion, Cesumar University (UniCesumar), Maringá 87050-900, Brazil; braulio.branco@unicesumar.edu.br (B.H.M.B.); marques.deborah@hotmail.com (D.C.d.S.M.); 3 Departamento de Pediatría Estomatológica, Facultad de Odontología, Universidad de Talca, Talca 3465548, Chile; vperez@utalca.cl (V.A.P.V.); natalia.martinez@utalca.cl (N.M.E.); jbotelho@utalca.cl (J.N.B.); 4 Programa de Doctorado en Ciencias Morfológicas, Facultad de Medicina, Universidad de La Frontera, Temuco 4811230, Chile; f.villagran04@ufromail.cl; 5 Escuela de Tecnología Médica, Facultad de Salud, Universidad Santo Tomás, Osorno 5310431, Chile; 6 Departamento de Medicina Interna, Facultad de Medicina, Universidad de La Frontera, Temuco 4811230, Chile; 7 Núcleo Científico y Tecnológico en Biorecursos (BIOREN), Universidad de La Frontera, Temuco 4811230, Chile; 8 Departamento de Enfermería, Fisioterapia y Medicina, Universidad de Almería, 04009 Almería, Spain; tpc468@ual.es; 9 Hospital Universitario Torrecárdenas, 04009 Almería, Spain

**Keywords:** autism, oral pathologies, stress measurement, scale validation

## Abstract

**Background/Objectives:** Research indicates that children with autism spectrum disorder (ASD) exhibit a deficiency in skills and initiative when it comes to adhering to daily oral hygiene routines. This, in turn, increases the likelihood of oral pathologies, thereby placing a significant emotional strain on their parents. In addition to the typical stress they already experience, parents of children with ASD are also burdened with pediatric oral health issues. However, a review of the literature reveals a lack of studies measuring stress in parents of children whose oral health affects their autistic condition. This research aimed to design and to validate a stress scale for parents of autistic children with poor oral hygiene. **Methods:** The study used an exploratory, non-experimental design to validate and determine the reliability of the scale. Scale reliability was assessed through a cross-sectional pilot test to evaluate internal scale consistency, with a focus on item similarity. We used Aiken’s V to estimate the validity of the scale, and Cronbach’s α for calculating scale reliability. Calculations, estimations, and statistical analysis were conducted using SPSS. **Results**: Expert validation, a pilot test, and a cross-sectional, non-experimental design established the reliability of the scale. **Conclusions**: We conclude that the 20-item scale exhibits validity (0.95) and reliability (0.965), ensuring its applicability in future research.

## 1. Introduction

The article focuses on oral hygiene-related stress in parents of children with ASD. Stress has been defined as a state or condition that involves worry and mental tension generated by a difficult situation, and manifests in how people react or respond naturally to threats and other stimuli [1]. Different models and theories have aimed to explain this state in humans, categorizing them into three classes: response-based, stimulus-based, and interaction-based [2].

According to interaction-based or transactional theories, in a typical family, the interaction between the father, mother, and children typically generates manageable levels of stress among parents. However, when the family has a child with a disability, parental stress levels rise significantly [3], as with parents of children with autism spectrum disorder (ASD). Specifically, studies report that the parents of autistic children repeatedly experience high stress levels, fundamentally linked to this disability [4,5,6,7,8,9,10,11,12,13,14,15,16].

Significant communication and social interaction difficulties, along with restricted and repetitive behaviors, are common autistic traits that contribute to high levels of parental stress [17]. These traits can significantly affect their independence and ability to fulfill daily cleanliness rituals in general and oral hygiene in particular [18,19,20]. However, in the scientific literature, no studies have specifically addressed the relationship between the oral hygiene of children with ASD and their parents’ stress levels. Given the identified gap in the literature, this research aims to design and validate a specifically customized scale to measure stress in parents of children with ASD, with a particular focus on the oral hygiene variable. This study also represents a novel contribution to the fields of psychometrics, autism research, and pediatric oral care. By designing and validating this scale, we aim to provide a valuable tool for future studies and diagnostic efforts to develop targeted intervention and coping programs for parents, regardless of their prior knowledge, socioeconomic status, or cultural background.

This study addresses a pressing need for a specialized assessment tool: can a reliable scale be developed and validated to measure stress in parents of autistic children with poor oral hygiene? As the next section demonstrates, this research is crucial due to a lack of such instruments in the current literature and the unique challenges faced by this population.

The literature has reported studies about measuring stress in the parents of children with disabilities and autism, as reported in a systematic review [21]. At the end of this article, there is also a particularly intriguing review of studies on designing and validating scales to measure stress, regardless of sample and context. Following up on this point, Becerra et al. [22] designed and validated an instrument to evaluate stress levels in physical therapy students carrying out their clinical practice. The researchers named this instrument “Estrigeree”. The researchers included constructs associated with stressful situations in its construction. Validation was then carried out via expert judgment, a test–retest pilot program with a sample of 10 subjects, and a final test with a sample of 100. The pilot program results showed a Cronbach’s α of 0.87 on the test and 0.62 in the retest. Researchers concluded that the instrument is valid and reliable for its temporal stability, along with being clear and complete. Even though this study did not specifically measure stress in the parents of disabled children, the study contributed to constructing instruments for examining stress. 

Nielsen et al. [23] determined the construct validity and psychometric properties of the Parental Stress Survey (PSS), which evaluates the perceived stress arising from the parental role. For validation, the models from Rash were used in a sample of parents with children between 2 and 18 years old, with and without behavioral disorders. The validation process reported that the PSS has two distinct subscales that measured parents’ stress on one side and their lack of satisfaction on the other. The authors concluded that a version of the PSS with 16 items, qualifying as two subscales, would be more suitable for parents of children with behavioral disorders compared to a sample unaware of such issues. However, the contribution to research from the study lies in the subscale evaluating stress, which serves as a model for specifically designing a scale to measure stress in parents of autistic children with poor oral hygiene. 

Another study used to model the procedure for determining instrument validity and reliability came from Talaee et al. [24], who validated an instrument to measure stress and fatigue among health care workers during the COVID-19 pandemic. To this end, they applied the content validity test, Cronbach’s α, and the test–retest reliability method for 60 health care workers. The content validity ratio of 46 questions, which was equal to 1, demonstrated significant validity and led to the inclusion of these items in the final survey version. All sections had content validity indices above 0.79 as well, confirming that the content was valid. For different survey sections, the Cronbach’s α ranged between 0.80 and 0.95, confirming the stability and high reproducibility of the survey. The researchers concluded that the analyzed instrument offered adequate validity and reliability levels for evaluating stress, anxiety, and depression amongst health care workers involved with the COVID-19 pandemic.

Tanta-Luyo et al. [25] designed and validated a stress coping scale for parents of children with disabilities to determine the psychometric evidence presented by this scale, specifically regarding the disability variable. The researchers applied a cross-sectional psychometric design to a sample of 246 parents and children in three special primary education institutions. Expert judgment and psychometric evaluation validated the content. The results reflect internal validity evidence, and the study concluded that the coping scale for parents of disabled children showed evidence of adequate psychometric properties. 

Mohammadi et al. [26] also created a psychometric scale to assess parental competence in parents of children with autism. This scale underwent content validity tests, leading to 12 items being removed. The validity ratio was 0.75, while the content validity index was 0.85. The Cronbach’s coefficient, which was 0.98 for the entire instrument, indicated reliability. The authors concluded that the parental competence scale exhibited acceptable psychometric properties, making it suitable for evaluating parental competence in parents of autistic children. By contrast with the preceding studies, Zhang et al. [27] adapted and validated an existing scale: the Parental–ASD Behaviour Scale. Chinese parents of autistic children adapted and applied the new scale, with results indicating that the adaptation presented acceptable reliability and validity. Finally, Desiningrum et al. [28] administered the Parental Stress Scale to parents of special needs children in Indonesia. Their results confirmed that the PSS had convergence and discriminating validity, along with being reliable for use in the Indonesian context. 

Based on limitations found in existing instruments and a clear absence of measurement tools combining key study variables, we hypothesize our developed scale will achieve high validity and reliability scores (Aiken’s V and Cronbach’s α exceeding 0.80) when measuring stress levels in parents of autistic children with poor oral hygiene. Results should effectively differentiate stress levels while maintaining consistency through test–retest reliability measures. A focused approach to oral hygiene challenges in autism promises to deliver an accurate, reproducible assessment instrument.

## 2. Materials and Methods

### 2.1. Validity and Reliability as Measurement Instrument Requirements

All measurement instruments must meet two essential requirements: validity and reliability [29]. Without meeting these needs, the instrument can present errors and biases that invalidate any research. The concept of instrument validity is straightforward and precise: an instrument is considered valid when it can accurately measure the variable that the study objective declares.

However, the matter presents various complications. Unlike measuring instruments from the natural sciences, in the social sciences it is possible that not every item on an instrument is pertinent or adequate to measure a variable or social construct. For instance, if we seek to measure “self-esteem”, it is possible that some items refer to other variables like “self-image” and “self-confidence”. In order to guarantee this validity requirement, it is necessary to apply procedures and tests to evaluate each item and the instrument overall. Even when there are various types of validity, one major type is content validation via expert judgment or external validators, accompanied by the Aiken V coefficient calculation, whose values appear in Table 1.

Instrument reliability or stability refers to the regularity or consistency of the results from the instrument. In other words, the instrument is reliable to the extent that the results are very similar or approximately similar after several measurements. Similar to validity, determining the reliability of a measuring instrument involves various procedures and tests. However, the most common method involves determining internal consistency and calculating the Cronbach coefficient, as shown in Table 2.

### 2.2. Research Design

The pilot study used an exploratory, non-experimental design to validate and determine the reliability of the scale. Application occurred during March 2024, utilizing the expert validation method for content validity. Scale reliability was assessed through a cross-sectional pilot test to evaluate internal scale consistency, with a focus on item similarity. A test–retest design, involving two separate applications, was then used to estimate the stability of the scale. Calculations, estimations, and data analysis were conducted using Excel spreadsheets (version 2019) and SPSS statistical software (IBM SPSS Statistics, Version 25, IBM Corp., Armonk, NY, USA).

There were ten fundamental research phases:

1. To conduct an exhaustive literature review to identify variables and indicators; and defining the constructs to incorporate in the measurement scale. The main models of instruments to measure parental stress that were used as examples for developing the proposed scale came from Abidin et al. [30], Freitas et al. [31], and Moreno [32].
2. The first version of the scale construction process involved drafting 20 items.
3. Validating the scale validation via the content validity test following 11 experts’ judgment.
4. Applying the Aiken V coefficient to determine agreement between judges or validators.
5. Correcting items as a function of their clarity and pertinence.
6. Applying the pilot test to a sample of parents of children with ASD who received the scale via email.
7. Calculating the internal reliability and consistency calculations using Cronbach’s α coefficient.
8. Applying the scale at two different times 15 days apart, i.e., test–retest. Both cases involved sending the scale via email.
9. Estimating scale stability by calculating the correlation between the scores obtained on the test and the retest, i.e., application at the first and second times.
10. The final scale version has undergone definitive correction and design.

### 2.3. Ethical Considerations for the Study

In this study, as with any other, we adhered to the following ethical principles:

1. Anonymization: guaranteed by not requesting participants’ names or any other type of personal identification.
2. The authors pledge to maintain confidentiality by not disclosing any specific or personal information about the sample members. In this case, we only show the statistically processed data and results.
3. When responding to the scale, sample members authorized their participation in the study and accepted the previously stated conditions, demonstrating informed consent.

### 2.4. Validation Participants

Eleven judges, who were autism experts with professional credentials and job experience, evaluated and validated the scale, providing evaluative judgments on the content of each item. Consequently, the expert sample selection was intentional and non-probabilistic, based on professional training criteria and their experience in the mental health and autism areas. We used a specially designed formula for this validation. We included the following criteria to validate each item:

1. Sufficiency refers to whether the content of each item is complete enough to measure the variable, specifically parental stress.
2. Coherence refers to how an item relates to other items and the context of the research.
3. Pertinence: The relevance of the items lies in their alignment with the topic and the variable under measurement.
4. Clarity: simple and precise item drafting, making it easily understandable.
5. Relevance pertains to the necessity and significance of each item in assessing the study object variable.

We used Aiken’s V to estimate the validity of the scale.

$$V=\frac{\bar{X}-l}{k}$$

where:

V = Aiken’s V coefficient.
$\bar{X}$ = average of all judges’ ratings.
l = the lowest possible score.
k = the range of possible values on the Likert scale used (difference between highest and lowest ratings).

### 2.5. Reliability Test Participants

We sought a pilot sample to apply the scale and verify internal consistency per item, as the scale targets parents of autistic children with deficient oral hygiene. We obtained this sample by contacting parents who regularly brought their children to an oral service specializing in children with ASD. The parents, reached via phone and email, provided informed consent to participate in the study.

Non-probabilistic convenience sample selection was carries out based on the fundamental inclusion criterion of having an autistic child with poor oral health diagnosed by the dentist. Overall, we used a non-probabilistic convenience method to choose the pilot sample, which is an excellent sampling strategy for exploratory and pilot studies.

The sample comprised 297 participants, comprising 249 mothers (females) and 48 fathers (males), with an average age ranging from 30 to 50 years. The marital status of the participants was evenly distributed, with approximately 50% married and the other half single. We observed an equal proportion for educational status, with 50% of the sample having at least undergraduate studies and the other half having a high school-level education. Finally, out of 297 participants, over 50% indicated that they were at a medium socioeconomic level, while the rest were from a lower stratum. 

Cronbach’s α was the formula for calculating scale reliability.

$$\alpha =\frac{K}{K-1}\left[1-\frac{\sum {S_{i}}^{2}}{{S_{T}}^{2}}\right]$$

where:

α = Cronbach’s Alpha coefficient.
K = number of items on the scale.
${S_{i}}^{2}$ = variance of each item.
${S_{T}}^{2}$ = variance of the scale.

## 3. Results

### 3.1. Scale Validation

Calculating Aiken’s V coefficient lets us quantitatively estimate the validity of evidence based on the content of items comprising a test, on the basis of the ratings obtained from expert judges. This coefficient has values between 0 and 1, with values closer to 1 indicating greater agreement between judges, ultimately demonstrating more content validity evidence. The following cutoff points were also considered for the validity scale.

Values below 0.5 = little agreement between evaluators; values between 0.5 and 0.74 = moderate agreement; and values above 0.75 = high agreement.

Table 3 displays the values obtained for Aiken’s V coefficient for each item and criterion considered, with a confidence interval above 75%. The lowest-scoring items in this area are P1, P4, and P5, with a general value of 0.8 and presenting clarity and sufficiency levels (0.5 points). In general, all of the criteria evaluated received a score above 0.8, indicating a satisfactory enough score from the evaluators to accept the validity of the instrument.

### 3.2. Scale Reliability Test

Reliability testing was conducted using the Cronbach’s α reliability test for a group of 279 subjects who answered the survey after expert validation. The results presented in Table 4 and Table 5 demonstrate a high reliability level of 0.965, suggesting that the instrument provides stable results and can effectively assess stress perceptions among parents of autistic children with inadequate oral hygiene.

Along with the Cronbach’s α result (Table 4), Table 5 shows the standardized Cronbach’s α values for each item, with the highest value possible being 0.966.

### 3.3. Test–Retest Results

The test–retest method was used twice for the scale on a group of 297 people with 15 days between tests, showing a strong and significant correlation between the two measurements (0.99), as Table 6 indicates. This statistical value complements the result of internal consistency (Cronbach’s α) and reinforces the general reliability of the designed scale. Consequently, the results for the reliability estimation of the present scale indicate that it easily satisfies the reliability level required by the scientific literature for this type of instrument. Table 7 displays the final validated scale following the completion of reliability tests.

## 4. Discussion

An exhaustive search confirmed the absence of studies regarding the stress levels experienced by parents due to the poor oral hygiene of their autistic children. In this sense, we also corroborated the non-existence of measuring instruments including scales and surveys for this specific situation, namely, measuring stress in the parents of children with ASD whose lack of oral hygiene creates a greater risk of suffering oral pathologies.

It appears that various disciplines, from health to social and behavioral sciences, need to consider oral hygiene and have a validated scale to measure stress in parents of children with ASD. This research makes significant contributions by providing a reliable instrument that can serve as a valuable tool for future studies and interventions in the field.

In general, the scale validation and reliability results align with other studies that constructed and validated scales. In particular, the validity and reliability coefficients found in this study are higher than those found by Becerra et al. [22]. They made a stress scale and reported a Cronbach’s α coefficient of 0.87, which is below our results of 0.96.

The results obtained in this study also approximate those from Talaee et al. [24], where the content validity index was above 0.79 with a Cronbach’s α coefficient ranging between 0.80 and 0.95. In our present study, total validity was 0.96 while reliability was 0.965. These results validate the validity and reliability of the scale, designed to measure stress levels in parents of autistic children with poor oral hygiene (Table 7), thereby meeting the necessary requirements for future studies.

There is also a satisfactory approximation to the results from Mohammadi et al. [26], whose content validity was 0.85 against 0.95 from the present study. There is also a satisfactory approximation for reliability, specifically the Cronbach’s α coefficient, with 0.965 in the proposed scale versus 0.98 obtained by Mohammadi et al. [26]. Once again, we confirm the rigorousness of the scale for measuring stress in parents of autistic children with deficient oral hygiene, which meets the basic needs of validity and reliability. Finally, the validity and reliability tests applied to the designed scale present results similar to those obtained by Zhang et al. [26] and by Desiningrum et al. [28]. In particular, the values reported in the latter work have a validity of 0.98 and a reliability of 0.92, which are similar to those found in the present study.

In short, a high Cronbach’s α coefficient means that the designed scale has reliable precision and replicability. The results obtained with this coefficient mean that its repeated application in similar samples will also yield similar and consistent results over time. The limitation of this test lies in its inability to apply to samples with different characteristics. For example, the validity and reliability tests applied to this scale do not guarantee its applicability, validity, or reliability for measuring other constructs, such as motivation and self-esteem.

Despite the impact of our present results, this study has recognizable limitations, including a shortage of background or prior studies about stress levels in parents of autistic children with deficient oral hygiene, leading to the non-existence of scales to measure this construct. However, this absence presented an opportunity to make relevant contributions to a problem that undoubtedly warrants further research and responses. Another limitation was the probable selection bias for participants, since these only came from the capital region, which may not reflect the diversity of the target population. We thus suggest expanding future research to other regions of Chile and other countries to broaden the study sample, its characteristics, and its diversity.

Among the precautions to consider when applying the scale are the ethical requirement of obtaining informed consent from parents and applying it in a calm environment without the presence of autistic children.

Overall, despite the lack of studies on the relationship between parental stress levels and the oral hygiene of their autistic children in the extant scientific literature, the reviewed studies on measuring parental stress provide valuable insights for developing a scale. Once validated, this scale can help initiate the first studies on this topic. Based on this, we can clearly distinguish the present study from others, particularly in its unique scale design that includes a new variable of significant importance and stress impact for parents of children with ASD: deficient oral hygiene, which can lead to oral pathologies.

## 5. Practical Implications of the Scale

The main utility and practical application of the scale is to diagnose stress levels. Parents of children with ASD who present high levels of stress have a high risk of worsening their mental health. Excessive anxiety and depression can cause disorders and imbalances that would prevent adequate care for their autistic children. In certain situations, an emotionally unstable parent would no longer be suitable to keep custody of their child. Applying the scale thus determines the stress levels, and the type of intervention required by the parents. Even if the scale reveals high levels of stress, it could serve as a tool for legally determining the custody of their children. On the other hand, if the scale reveals an accurate diagnosis, parents can implement treatments that will impact quality of life for themselves and for their children with ASD.

## 6. Conclusions

The scale created to measure stress levels in the parents of autistic children with oral hygiene problems represents an important advancement in the research field. The literature focusing on this specific aspect has not yet produced a similar tool. The present study is a significant contribution to various disciplines, including occupational therapy, pediatric dentistry, family psychology, and psychopedagogy, given that it provides a validated and unique instrument to evaluate this underexplored phenomenon.

The scale meets the essential validity and reliability requirements of any measurement scale. Extensive tests to validate and adjust the proposed scale supported the methodological rigor of the study, resulting in exceptionally high scale validity and reliability. Regardless of their geographical, cultural, or socioeconomic context, health professionals can confidently use the scale in specific populations of parents whose children have ASD and present oral hygiene problems, as it serves as a highly useful tool for creating guided interventions for both autistic children and their parents. Regarding the cultural factors that could influence the application of the scale, such as customs, traditions, and hygiene habits, these would be isolated, and their impact would be almost zero since the construct to be measured is the level of stress caused by having an autistic child, regardless of culture. However, it is evident that culture can influence stress levels, and future research should consider this variable.

We recommend pursuing further research on the stress levels of parents of autistic children, as there are numerous unexplored areas in this field. Developing new evaluation tools and gaining a deeper understanding of the factors influencing these parents’ stress can have a significant impact on the quality of life for both children with ASD and their parents. Future studies could adapt the scale to other disabilities beyond autism. We suggest applying it to parents of Down syndrome and cerebral palsy cases. Longitudinal studies could also apply it to track the evolution of parents’ stress levels, and their variations based on intervention programs.

## Figures and Tables

**Table 1:** Validity coefficient interpretation.

Coefficient	Validity Level
0.91 to 1.00	Very high
0.81 to 0.90	High
0.71 to 0.80	Moderate
0.61 to 0.70	Low
0.51 to 0.60	Very low

**Table 2:** Interpretation of Cronbach’s α coefficient.

Coefficient	Reliability Level
0.81 to 1.00	Very high
0.61 to 0.80	High
0.41 to 0.60	Moderate
0.21 to 0.40	Low
0.01 to 0.20	Very low

**Table 3:** Scale validity: values obtained for Aiken’s V coefficient on each item and criterion considered.

Items	Sufficiency	Pertinence	Coherence	Clarity	Relevance	Total
P1	0.91	0.95	0.91	0.68	0.95	0.88
P2	0.95	0.95	1.00	0.86	1.00	0.95
P3	0.95	0.95	1.00	1.00	1.00	0.98
P4	0.77	0.95	1.00	0.68	0.95	0.87
P5	0.82	0.95	1.00	0.68	0.95	0.88
P6	0.91	0.86	1.00	0.91	1.00	0.94
P7	1.00	0.95	1.00	0.91	0.95	0.96
P8	0.95	1.00	1.00	0.95	0.91	0.96
P9	1.00	0.95	1.00	1.00	1.00	0.99
P10	0.82	0.95	1.00	1.00	1.00	0.95
P11	0.86	0.91	1.00	1.00	0.95	0.95
P12	1.00	0.95	1.00	1.00	0.95	0.98
P13	0.95	0.91	1.00	1.00	1.00	0.97
P14	1.00	0.95	1.00	1.00	1.00	0.99
P15	0.95	0.91	1.00	1.00	0.91	0.95
P16	1.00	0.95	1.00	1.00	0.95	0.98
P17	1.00	0.95	1.00	1.00	0.95	0.98
P18	0.95	0.95	1.00	1.00	1.00	0.98
P19	1.00	0.95	1.00	1.00	0.91	0.97
P20	0.95	0.95	1.00	1.00	1.00	0.98
Total	0.94	0.95	1.00	0.93	0.97	0.96

**Table 4:** Scale reliability according to internal consistency.

Cronbach’s Alpha	Cronbach’s α Based on Standardized Elements	Number of Items
0.965	0.966	20

**Table 5:** Total element statistics.

Statements Evaluated	Median on Scale If Element Is Suppressed	Scale Variance If Element Is Suppressed	Total Correlation of Corrected Elements	Multiple Correlation Squared	Cronbach’s Alpha If Element Is Suppressed
**P1**	64.70	274.530	0.796	0.896	**0.963**
**P2**	64.45	271.818	0.769	0.887	**0.963**
**P3**	64.82	264.841	0.846	0.885	**0.962**
**P4**	64.42	271.002	0.867	0.903	**0.962**
**P5**	64.79	272.860	0.859	0.946	**0.962**
**P6**	64.27	274.205	0.832	0.920	**0.962**
**P7**	65.03	272.905	0.690	0.842	**0.964**
**P8**	65.27	273.580	0.661	0.815	**0.964**
**P9**	64.70	268.530	0.776	0.903	**0.963**
**P10**	64.79	271.422	0.801	0.898	**0.963**
**P11**	64.58	270.939	0.828	0.906	**0.962**
**P12**	64.45	271.381	0.802	0.860	**0.963**
**P13**	64.36	275.926	0.563	0.853	**0.966**
**P14**	64.42	276.689	0.741	0.920	**0.963**
**P15**	64.39	274.121	0.676	0.930	**0.964**
**P16**	64.73	277.142	0.692	0.813	**0.964**
**P17**	64.97	272.030	0.796	0.869	**0.963**
**P18**	65.94	282.809	0.497	0.603	**0.966**
**P19**	64.64	270.176	0.875	0.931	**0.962**
**P20**	64.58	272.814	0.714	0.763	**0.964**

P1 to P20 (bold): a series of statements used to evaluate the level of stress in parents of autistic children with poor dental hygiene; the Cronbach’s alpha for the entire scale (if that statement is removed) is shown in the last column.

**Table 6:** Correlations between scores obtained on the first and second times.

		First Application	Second Application
First application	Pearson’s Correlation	1	0.999 **
	Sig. (bilateral)		0.000
	**N**	**297**	**297**
Second application	Pearson’s Correlation	0.999 **	1
	Sig. (bilateral)	0.000	
	**N**	**297**	**297**

** Correlation is significant at the 0.01 level (bilateral). The bold indicates the total number of participants involved in the reliability test.

**Table 7:** Definitive validation scale after undergoing reliability tests.

Stress Levels: Very Low (1); Low (2); Medium (3); High (4); Very High (5)						
		1	2	3	4	5
1	The lack of oral hygiene in my child makes my stress level					
2	Knowing that my child can get some type of oral disease due to poor oral hygiene makes my stress level					
3	Not knowing about the consequences of a lack of oral hygiene in my child makes my stress level					
4	The challenges my child faces in properly carrying out oral hygiene makes my stress level					
5	Being aware of the efforts my child makes to perform proper oral hygiene makes my stress level					
6	My child presenting signs of oral diseases makes my stress level					
7	Assisting my child in their oral hygiene routine (brushing) makes my stress level					
8	Observing my child during toothbrushing makes my stress level					
9	Not assisting my child during their oral hygiene makes my stress level					
10	Seeing that my child cannot independently carry out their oral hygiene makes my stress level					
11	Not seeing any progress from my child in their oral hygiene skills makes my stress level					
12	The resistance from my child against oral hygiene routines (brushing) makes my stress level					
13	Taking my child to oral appointments makes my stress level					
14	Considering that my child can be a target for bullying due to their lack of oral hygiene makes my stress level					
15	Not taking my child to dentists’ visits makes my stress level					
16	Not being able to act when my child is bullied due to their lack of oral hygiene makes my stress level					
17	Controlling the oral hygiene of my child makes my stress level					
18	Receiving support to improve the oral hygiene of my child makes my stress level					
19	Not having control over the oral hygiene of my child makes my stress level					
20	Not getting support to improve the oral hygiene of my child makes my stress level					

## Data Availability

Data is contained within the article and Supplementary Material.

## Supplementary Materials

The following supporting information can be downloaded at: https://www.mdpi.com/article/10.3390/healthcare12222215/s1, Scale validation—Aiken V.

## References

[1] World Health Organization (2023). Stress. https://www.who.int/es/news-room/questions-and-answers/item/stress.

[2] Larzabal-Fernández A., Ramos-Noboa M. (2019). Propiedades psicométricas de la escala de estrés percibido (pss-14) en estudiantes de bachillerato de la provincia de Tungurahua (Ecuador). Ajayu.

[3] Hsiao Y.J. (2018). Parental Stress in Families of Children with Disabilities. Interv. Sch. Clin..

[4] Al-Oran H.M., Khuan L. (2021). Predictors of parenting stress in parents of children diagnosed with autism spectrum disorder: A scoping review. Egypt J. Neurol. Psychiatry Neurosurg..

[5] Azamin M., Zakaria R., Zainal M., Izzati N. (2022). Parental Stress and Coping Attitudes in Autism Spectrum Disorder Children: A Survey during Movement Control Order period amid COVID-19 Pandemic. Malays. J. Med. Health Sci..

[6] Baker-Ericzén M.J., Brookman-Frazee L., Stahmer A. (2005). Predictors of parenting stress in parents of children diagnosed with autism spectrum disorder: A scoping review. Res. Pract. Pers. Sev. Disabil..

[7] Enea V., Rusu D.M. (2020). Raising a Child with Autism Spectrum Disorder: A Systematic Review of the Literature Investigating Parenting Stress. J. Ment. Health Res. Intellect. Disabil..

[8] Curley K., Colman R., Rushforth A., Kotera Y. (2023). Stress Reduction Interventions for Parents of Children with Autism Spectrum Disorder: A Focused Literature Review. Youth.

[9] Di Renzo M., Guerriero V., Petrillo M., Bianchi di Castelbianco F. (2022). What is Parental Stress Connected to in Families of Children with Autism Spectrum Disorder? Implications for Parents’ Interventions. J. Fam. Issues..

[10] Galpin J., Barratt P., Ashcroft E., Greathead S., Kenny L., Pellicano E. (2018). ‘The dots just don’t join up’: Understanding the support needs of families of children on the autism spectrum. Autism.

[11] Jolly K., Huber T. (2020). Prevalence of Anxiety and Stress in Mothers of Children with Autism Spectrum Disorder (ASD): A Review of Literature. Res. J. Educ..

[12] Ng C.S.M., Fang Y., Wang Z., Zhang M. (2021). Potential Factors of Parenting Stress in Chinese Parents of Children with Autism Spectrum Disorder: A Systematic Review. Focus Autism Dev. Disabil..

[13] Ni’matuzahroh, Suen M.W., Ningrum V., Widayat, Yuniardi M.S., Hasanati N., Wang J.H. (2022). The Association between Parenting Stress, Positive Reappraisal Coping, and Quality of Life in Parents with Autism Spectrum Disorder (ASD) Children: A Systematic Review. Healthcare.

[14] Pratesi C.B., Garcia A.B., Pratesi R., Gandolfi L., Hecht M., Nakano E.Y., Zandonadi R.P. (2021). Quality of Life in Caregivers of Children and Adolescents with Autistic Spectrum Disorder: Development and Validation of the Questionnaire. Brain Sci..

[15] Porter N., Loveland K. (2019). An Integrative Review of Parenting Stress in Mothers of Children with Autism in Japan. Int. J. Disabil. Dev. Educ..

[16] Alegría P.L., Landim S.F., Branco B.H.M., Carmine F., Birditt K., Sandoval C., González M.M. (2024). Dental Hygiene Challenges in Children with Autism: Correlation with Parental Stress: A Scoping Review. J. Clin. Med..

[17] Lievore R., Lanfranchi S., Mammarella I.C. (2023). Parenting stress in autism: Do children’s characteristics still count more than stressors related to the COVID-19 pandemic?. Curr. Psychol..

[18] Bernath B., Kanji Z. (2021). Exploring barriers to oral health care experienced by individuals living with autism spectrum disorder. Can. J. Dent. Hyg..

[19] Como D.H., Stein Duker L.I., Polido J.C., Cermak S.A. (2021). Oral Health and Autism Spectrum Disorders: A Unique Collaboration between Dentistry and Occupational Therapy. Int. J. Environ. Res. Public Health.

[20] Erwin J., Paisi M., Neill S., Burns L., Vassallo L., Nelder A., Facenfield E., Devalia U., Vassallo T., Witton R. (2022). Factors influencing oral health behaviours, access and delivery of dental care for autistic children and adolescents: A mixed-methods systematic review. Health Expect..

[21] Agarwal R., Wuyke G., Sharma U., Burke S., Howard M., Li T., Sanchez M., Bastida E. (2024). Stress and Anxiety Among Parents of Transition-Aged Children with Autism Spectrum Disorder: A Systematic Review of Interventions and Scales. Rev. J. Autism Dev. Disord..

[22] Becerra M.G., Gómez M.V., Lugo R.I., Hernández G.A. (2018). Diseño y Validación del Instrumento Estrigeree para la evaluación del estrés en prácticas clínicas de fisioterapia. Eur. Sci. J..

[23] Nielsen T., Pontoppidan M., Rayce S.B. (2020). The Parental Stress Scale revisited: Rasch-based construct validity for Danish parents of children 2–18  years old with and without behavioral problems. Health Qual. Life Out..

[24] Talaee N., Varahram M., Jamaati H., Salimi A., Attarchi M., Kazempour M., Sadr M., Hassani S., Farzanegan B., Monjazebi F. (2022). Stress and burnout in health care workers during COVID-19 pandemic: Validation of a questionnaire. J. Public Health.

[25] Tanta-Luyo A.Y., Quispe Fernández M., Serpa Barrientos A., Ardiles Guevara D.E. (2020). Diseño y validación de la escala de afrontamiento al estrés en padres de hijos con discapacidad. Rev. Investig. Psicol..

[26] Mohammadi F., Rakhshan M., Molazem Z., Zareh N., Gillespie M. (2019). Development of parental competence scale in parents of children with autism. J. Pediatr. Nurs..

[27] Zhang C., Zhou T., Yi C., Liu S., Hong Y., Zhang Y. (2022). Adaptation and validation of the Parental Behaviour Scale for Autism Spectrum Disorder in Chinese parents. Res. Autism Spectr. Disord..

[28] Desiningrum D.R., Hermawati D., Somantri M., Indriana Y., Rusydana A. (2024). Parental stress scale validation. Asian J. Eng. Soc. Health.

[29] Mohamad M.M., Sulaiman N.L., Sern L.C., Salleh K.M. (2015). Measuring the Validity and Reliability of Research Instruments. Procedia Soc. Behav. Sci..

[30] Abidin R., Flens J.R., Austin W.G., Archer R.P. (2006). The Parenting Stress Index. Forensic Uses of Clinical Assessment Instruments.

[31] Freitas P.M., Barreto G.V., Teodoro M.L.M., Haase V.G. (2021). Questionário de estresse para pais de crianças com transtornos do desenvolvimento: Validação. Aval. Psicol..

[32] Moreno E. (2017). Instrumentos de Evaluación y Variables de Estrés en Padres y Madres de Niños con Autismo. Revisión y Líneas Futuras de Investigación para la Promoción de Salud Mental.

